# Psychometric Evaluation of the Making it CLEAR Questionnaire: A Resilience Measure for Older Adults

**DOI:** 10.1093/geroni/igab030

**Published:** 2021-08-13

**Authors:** Lucy Whitehall, Sylwia Górska, Robert Rush, Anusua Singh Roy, Linda Irvine Fitzpatrick, Kirsty Forsyth

**Affiliations:** 1 School of Health Sciences, Queen Margaret University, Edinburgh, UK; 2 Edinburgh Health and Social Care Partnership, NHS Lothian, UK

**Keywords:** Acute/short-term care, Frailty, Measurement, Resilience

## Abstract

**Background and Objectives:**

Previous efforts to develop a resilience measure for older adults have largely failed to consider the environmental influences on their resilience, and have primarily concentrated on the resilience of community-dwelling older adults. Our objective was to validate a new multidimensional measure of resilience, the Making it CLEAR (MiC) questionnaire, for use with older adults at the point of discharge from hospital.

**Research Design and Methods:**

This study tested the structure, validity, and reliability of the MiC questionnaire. The questionnaire consists of 34 items, which assess the “individual determinants of resilience” (IDoR) and the “environmental determinants of resilience” (EDoR) across 2 subscales. 416 adults aged 66–102 years participated. Exploratory factor analysis, item analysis, and linear regression were undertaken.

**Results:**

The IDoR subscale contained six factors which were labeled “Self-efficacy,” “Values,” “Interpersonal skills,” “Life orientation,” “Self-care ability,” and “Process skills.” The EDoR subscale contained five factors related to “Person–environment fit,” “Friends,” “Material assets,” “Habits,” and “Family.” Both subscales demonstrated acceptable convergent validity and internal consistency, while individual items showed acceptable levels of discrimination and difficulty.

**Discussion and Implications:**

The study provides evidence supporting the validity and quality of the MiC questionnaire. The results suggest that the MiC questionnaire could be used to identify the resilience needs of older adults at the point of hospital discharge. However, future research should identify which items of the MiC questionnaire are associated with hospital readmission, in order to develop an easily applicable screening tool for clinical practice.

**Translational Significance:** The Making it CLEAR questionnaire is a new measure, which assesses the multidimensional influences on the resilience of older adults. Specifically, the inclusion of environmental determinants of resilience makes this a novel and comprehensive tool, particularly as the influence of environmental factors on resilience is likely to be increased in advanced age. The Making it CLEAR questionnaire is intended for use by multidisciplinary clinical teams to identify older adults who may struggle to “adapt well” following acute hospital admission. The tool has the potential to enable the development of evidence-based resilience interventions, thus supporting complex decision making and personalized care.

## Background and Objectives

Resilience, or the process of effectively adapting to and managing adversity, is a construct that has been examined in multiple populations of older adults (Windle et al., 2011). These studies have found that a high level of resilience is protective against both mental and physical illnesses and is closely associated with overall well-being (Lu et al., 2017; Scelzo et al., 2018). Consequently, resilience is assumed to have a strong impact on patient health (Dong et al., 2013). However, these studies have primarily recruited community-dwelling older adults (Hardy et al., 2004; Windle et al., 2010). This is problematic given the contextual nature of resilience, which makes generalization of resilience research findings across populations a cause for concern (Hardy et al., 2004; Windle et al., 2010).

Nevertheless, self-reported resilience scales have been developed, with the aim of identifying older adults with low resilience who may be at risk of negative health outcomes (Hardy et al., 2004; Hicks & Conner, 2014). The implication is that these individuals could be identified and targeted resilience interventions could be developed to reduce their risk of negative health outcomes (Dong et al., 2013).

A methodological review of resilience measures by Windle and colleagues (2011) evaluated the quality and psychometric properties of 15 resilience measures. It concluded that the Connor–Davidson Resilience Scale, the Resilience Scale for Adults, and the Brief Resilience Scale were the most robust resilience measures. However, as the impact of environmental factors on individual’s resilience was routinely overlooked, no “gold standard” was found (Windle et al., 2011). These conclusions were supported by Cosco and colleagues (2016); who compared the psychometric properties of five resilience measures with samples aged 60+; each assessed resilience solely at the individual level at the expense of the environment.

This is problematic as environmental determinants have been recognized to influence the resilience of older adults (Windle et al., 2011). Numerous meta-analyses, systematic reviews, and empirical studies have identified both individual and environmental factors that influence older adults’ resilience, and are summarized in Table 1 (Bolton et al., 2016; Freitag & Schmidt, 2016; Górska et al., 2021; Hardy et al., 2004; Hayman et al., 2017; Hildon et al., 2008; Martin et al., 2015; Polson et al., 2018; Wells, 2010; Windle et al., 2008). Nevertheless, previous resilience measures have failed to incorporate items which reflect the environmental determinants of resilience (EDoR).

**Table 1.** Individual and Environmental Factors Which Influence the Resilience of Older Adults

Overarching theme	Protective factors	Vulnerability factors
Within the older adult		
Sociodemographic resources	Lower age Higher level of education Income	
Self-perceptions	Strong self-efficacy Sense of coherence Self-transcendence High self-esteem Self-acceptance Good self-rated health	Perceived stressfulness of the event/severity of the condition Poor self-rated health
Psychological resources	Positive emotions/happiness Optimism Emotional regulation Altruism Grit Hope Morale Satisfaction in life	Depression/depressive symptoms Psychological distress Anxiety Stress
Cognitive abilities	Cognitive functioning Communication skills	
Health status/behaviors	Good mental and physical health Independence in ADLs and mobility Meaningful activity Health-promoting lifestyle/self-care Successful ageing	ADL impairment Frailty
Previous adversities	Previous experience of overcoming adversity	Childhood adversity
Meaningfulness	Spiritual practice/being religious Meaning/purpose in life “Counting blessings”	
Within relationships		
Social support network	External connections Social support Social connectedness Social engagement Social network size	Loneliness
Family	Close family relationships Living with others	Being childless/limited support from children
Friends	Close friendships Neighbors	
Within the community		
Person–environment fit	“Places for growing older” Community involvement Social and economic resources Health care and agencies	

*Note*: ADL = activities of daily living.

Consequently, it has been recommended that a resilience measure that captures all relevant factors is developed, in order to provide a robust evaluation of older adults’ resilience and to facilitate the development of resilience interventions (Górska et al., 2021; Windle et al., 2011). In recognition of this, two multidimensional resilience measures for older adults have been developed. These are the Multidimensional Individual and Interpersonal Resilience Measure (MIIRM; Martin et al., 2015) and the Making it CLEAR (MiC) questionnaire (Queen Margaret University [QMU] and NHS Lothian, 2015).

In 2015, Martin and colleagues published the MIIRM. The MIIRM was developed to assess those family and individual factors related to resilience in older adults; however, the authors note a number of limitations. For instance, some wording is specific to the United States and would need adapting for international use (Martin et al., 2015). Furthermore, significant protective factors pertaining to health, functioning, and the physical environment are not measured, which may negatively impact the MIIRM’s capacity to adequately explore the complex dimensions of resilience.

The MiC (Community Living, Enablement, and Resilience) questionnaire was developed to comprehensively assess older adult’s perceptions of their resilience, based on examination of a range of factors occurring at the individual (e.g., determination, positivity and optimism, self-efficacy, and values) and environmental levels (e.g., family support, involvement in, and quality of, social networks, and ability to find and use social or community resources; QMU and NHS Lothian, 2015).

The first iteration of the MiC questionnaire consisted of 46 items, each pertaining to a factor associated with the resilience of older adults, which were identified through an integrative literature review (QMU and NHS Lothian, 2015). This 46-item MiC questionnaire was then piloted with 198 community-dwelling older adults. Psychometric analysis confirmed that this questionnaire had satisfactory construct validity, internal consistency, concurrent validity, and test–retest reliability (QMU and NHS Lothian, 2015). However, 22 items were found not to contribute to accurate measurement, and many were found to have poor discriminatory power as they tended to generate agreement.

In response to this analysis, the 22 redundant items were removed and 10 more difficult items were added; the wording of some of the retained items was also amended to make the items more difficult to endorse (i.e., through phrases such as “I can always…” and “I have no…”; QMU and NHS Lothian, 2015). The resulting MiC questionnaire consists of 34 items, split across two subscales, which assess the individual and environmental determinants of older adults’ resilience.

In comparison to the MIIRM, the MiC questionnaire includes three items that are related to the individual’s perceived health and three items related to the physical environment (see Tables 3 and 5 for the individual items of the MiC questionnaire). It could be concluded that the MiC questionnaire provides a more comprehensive evaluation of older adults’ resilience (QMU and NHS Lothian, 2015). However, the psychometric properties of the 34-item MiC questionnaire have not yet been assessed.

Given that receipt of acute hospital care may diminish the psychological resources of older adults (Whitehall et al., 2020), the aim of this study was to validate the current iteration of the MiC questionnaire with a population of older adults ready for discharge from a medicine of the elderly (MoE) ward.

## Research Design and Methods

### Participants and Sample Size Justification

This study used data collected as part of the MiC-MoE study, a prospective study investigating the resilience of older adults ready for discharge from a MoE ward, and its association with hospital readmission. The MiC-MoE sample was recruited from three MoE wards in a 900-bed, urban acute teaching hospital over a 13-month period, from August 2018 to September 2019. MoE wards are defined as those providing 24-hr, acute, medical, and multidisciplinary care for older adult patients (>65 years) admitted to hospital with a frailty syndrome (e.g., delirium, immobility, falls); patient stays typically exceed 48 hr (Baxter et al., 2018; Lyndon et al., 2014).

Patients were considered eligible for inclusion in this study if they were aged 65 or older, had capacity to provide written informed consent and to understand and respond to questions in the English language, were medically fit to participate, and were ready for discharge, which was defined as being “assessed by the medical team responsible for their care as medically fit to be discharged back to their original place of residence.”

Each participant provided informed written consent prior to data collection, and consented to their data being used in secondary analyses. A detailed diagram of all the steps taken to implement the MiC-MoE study and the participant information leaflet are provided in Supplementary Figures A and B, respectively. Ethical approval for the study was granted by the North West—Lancaster Research Ethics Committee (reference number: 16/NW/0077) and the NHS Lothian Research and Development office (project ID: 2006/0025).

Four hundred nineteen participants were recruited for the MiC-MoE study; of these, 416 were included in this validation study as three participants did not complete the MiC questionnaire. This sample size was satisfactory for exploratory factor analysis (EFA), as it is advised that EFA should use data from at least 300 participants, or should allow for 5–10 observations per variable (Comrey & Lee, 1992; Yong & Pearce, 2013). Given that the largest subscale of the MiC questionnaire contains 21 items, a sample size of 416 met these requirements.

### Instruments

#### The MiC questionnaire

The MiC questionnaire contains 34 items addressing a variety of factors understood to influence older adults’ resilience (QMU and NHS Lothian, 2015). These items are split across two distinct subscales, one assessing the individual determinants of resilience (IDoR), which consists of 21 items, and one assessing the EDoR, which consists of 13 items. Items address participants perceptions of their self-care, leisure, work, responsibilities, social environment, resources, habits, values, self-efficacy, motor skills, communication skills, and process skills (QMU and NHS Lothian, 2015).

Participants are asked to rate their level of agreement for each item on a 4-point scale (i.e., strongly agree, agree, disagree, and strongly disagree). For each item, 0–3 points were given on the basis of level of agreement such that higher scores indicated stronger agreement.

The IDoR subscale has a maximum score of 63; descriptive interpretation of scores is as follows: 0–21 = poor IDoR, 22–42 = moderate IDoR with some areas of need, and >43 = high IDoR (QMU and NHS Lothian, 2015). The EDoR subscale has a maximum score of 39; scores are interpreted as follows: 0–13 = poor EDoR, 14–26 = moderate EDoR with some areas of need, and >27 = high EDoR (QMU and NHS Lothian, 2015).

#### Clinical Frailty Scale

The Clinical Frailty Scale (CFS) is a 9-point scale which broadly assesses frailty based on the clinical health and performance abilities of the older adult (Rockwood et al., 2005). The CFS has good concurrent validity with the 70-item Frailty Index (*r* = .8), and has been validated as an adverse outcome predictor for older adults hospitalized with acute illness, such outcomes include in-hospital mortality, care home placement, and length of stay (Basic & Shanley, 2015; Rockwood et al., 2005).

As such, the CFS is beginning to be routinely used in hospital settings, particularly as it is quick to complete and does not require extra staff, the measurement of specific items, or use of specialized equipment (Conroy & Dowsing, 2013; Martocchia et al., 2013). These characteristics also make it appropriate for research conducted in acute hospital wards.

#### Optum SF-12v2 Health Survey

The Optum SF-12v2 Health Survey (Ware et al., 2009) is a patient-reported multidimensional measure of functional health and well-being. It consists of 12 items covering eight health domains: Physical Functioning, Role—Physical, Bodily Pain, General Health, Vitality, Social Functioning, Role—Emotional, and Mental Health. Based on an individual’s response to each item, composite scores are produced for the Physical Component Summary (PCS) and Mental Component Summary (MCS) scales (Ware et al., 2009). Composite scores range from 0 to 100, where a zero score indicates the lowest level of health and 100 indicates the highest level of health (Ware et al., 2009). These scores have been shown to reflect the PCS and MCS scale values obtained by the 36-item Short-Form Health Survey (McDowell, 2006).

### Procedure

After providing informed consent, participants were provided with a paper copy of the MiC questionnaire and the Optum SF-12v2 Health Survey. As both the MiC questionnaire and the Optum SF-12v2 Health Survey are self-report measures, participants were asked to complete them independently. However, if assistance was required with completing the questionnaires, a member of the research team would support the participant. Participants were not supported by a family member or a member of their clinical team, and this reduced the risk of response bias in this study.

The CFS score was completed by a consultant geriatrician responsible for the care of the participant. Prior to the data collection period of the MiC-MoE study, the CFS was introduced as a part of the routine assessment of MoE patients during admission to the hospital. Consequently, the geriatricians who supported this study were using the tool as part of their routine practice were asked to rescore it at the point of discharge for the purpose of this study.

### Data Analysis

Data from the completed questionnaires were entered in a Microsoft Excel worksheet for electronic storage and quality checking. Data analysis for this study was conducted using R (R Core Team, 2018).

EFA was used to assess the factor structure of the MiC questionnaire subscales. Kaiser–Meyer–Olkin (KMO) measures and Bartlett’s test of sphericity were used to determine whether the data for the two MiC questionnaire subscales were suitable for EFA (Field et al., 2012). Scree plots and parallel analysis were used to inform the number of factors to extract for each subscale (Field et al., 2012).

As the factors measured by each subscale were assumed to correlate with one another, oblique rotation was performed (Field et al., 2012). Items with factor loadings greater than .4 were considered to load on a particular construct (Stevens, 2002). Items were identified as cross-loading if they loaded at .4 or higher on more than a single factor. The models were judged to have a good fit based on the criteria of a Tucker–Lewis Index (TLI) value greater than .9, the results of the parallel analysis, and whether the model was theoretically interpretable (Clark & Bowles, 2018). Discriminant validity was also assessed using a factor correlation matrix, to ensure that each factor assessed a unique variable—a correlation greater than .7 is indicative of poor discriminant validity (Stevens, 2002). 

Following the EFA, Cronbach’s α was used to assess the internal consistency of the subscales (de Vaus, 2002), while mean inter-item correlations were calculated to assess consistency within the factors (Tavakol & Dennick, 2011). Item total correlations (*r*) and item difficulty values were also calculated to determine whether individual items could discriminate between those who had a low IDoR or EDoR level, and those who had high levels (Nunnally & Bernstein, 1994; PearsonVue, 2015).

Finally, in order to assess convergent validity, the relationships between the MiC questionnaire subscales and frailty (measured by the CFS) and perceived physical and mental health (measured by the Optum SF-12v2 Health Survey) were assessed using linear regression.

Due to small sample sizes in four of the CFS categories, some categories were combined to avoid redundant levels (Wielenga, 2007). The categories “well” and “managing well” were combined under the title “managing well,” while the categories “severely frail” and “very severely frail” were combined under the title “severely frail.”

## Results

### Descriptive Analysis

The mean age of the participants (*n* = 416) was 85.33 (*SD*: 6.54, range: 66–102) years and 67.8% (*n* = 282) were female. Table 2 describes further sociodemographic characteristics of the participants included in the present study. The mean number of days between recruitment into the MiC-MoE study and hospital discharge was 1.47 days (*SD*: 2.59).

**Table 2.** Sociodemographic Characteristics of Participants (*n* = 416)

Variables	Mean	*SD*, range	Frequency	%
Continuous variable				
Age	85.33	6.54, 66–102		
Categorical variables				
Gender				
Male			134	32.21
Female			282	67.79
Marital status				
Married			86	20.67
Divorced			27	6.49
Single			19	4.57
Widowed			269	64.66
Separated			10	2.40
Never married			5	1.20
Ethnicity				
White			413	99.28
Mixed/multiple ethnic background			1	0.24
African, Caribbean, or Black			1	0.24
Asian			1	0.24
Religion				
Christian			229	55.05
No religion			164	39.42
Other			18	4.33
Declined to answer			4	0.96
Unknown			1	0.24
Living arrangement				
Lives alone			292	70.19
Lives with others			124	29.81
Location of residence				
Private residence—own home			241	57.93
Private residence—other			87	20.91
Supported accommodation			80	19.23
Nursing home			8	1.92

The mean IDoR subscale score was 43.2 (*SD*: 7.92, range: 24–63), while the mean EDoR subscale score was 24.94 (*SD*: 5.15, range: 11–39). The mean PCS score was 31.85 (*SD*: 9.33, range: 8.6–59.53) and the mean MCS score was 48.81 (*SD*: 8.95, range: 17.79–68.59).

Regarding CFS scores, 2.2% of participants were rated “managing well” (*n* = 9), 14.2% of participants were rated “vulnerable” (*n* = 59), 28.8% were rated “mildly frail” (*n* = 120), 45.4% of participants were rated “moderately frail” (*n* = 189), 7.9% of participants were rated “severely frail” (*n* = 33), and 0.7% of participants were rated “well” and “very severely frail” (*n* = 3 for both categories).

### EFA of the IDoR Subscale

EFA with promax rotation was performed on the data to explore the structure of the IDoR subscale. The data were deemed suitable for EFA based on a KMO value of .92 (Kaiser, 1974) and Bartlett’s test of sphericity, which indicated that correlations between items were sufficient (χ ^2^ (210) = 4196.994 [*p* < .001]).

Parallel analysis suggested that six factors should be extracted, while inflexions in the scree plot suggested five or six factors (Supplementary Figure C). Accordingly, the loadings of five- and six-factor solutions were estimated and examined.

The five-factor solution returned a TLI value of .898; a value lower than .9 is indicative of underfactoring and suggests that more factors are required (Clark & Bowles, 2018). The six-factor solution was, therefore, preferred with a TLI value of .922 and theoretically interpretable factors. Based on the content of high loading items, these factors were labeled “Self-efficacy,” “Values,” “Interpersonal skills,” “Life orientation,” “Self-care ability,” and “Process skills” (Table 3). This model accounted for 56% of the common variance.

**Table 3.** Six-Factor Solution for the “Individual Determinants of Resilience” Subscale

Item	Factor 1	Factor 2	Factor 3	Factor 4	Factor 5	Factor 6
	Self-efficacy	Values	Interpersonal skills	Life orientation	Self-care ability	Process skills
“I am physically able to do the things I need and want to”	.92					
“I am able to do things on my own”	.85					
“I always have enough energy to do the things I need and want to”	.81					
“I see myself as a healthy person”	.67					
“I feel in control of my life”	.49					
“I am a patient person”		.63				
“I find it easy to accept whatever life throws at me”		.58				
“I can forgive myself and others”		.57				
“I am generally happy”		.49				
“I can see the funny side of life”		*.38*				
“I have things to look forward to”		*.31*				
“I have no problems getting along with others and making new friends”			.81			
“I can always make myself understood to others”			.71			
“I am happy to help my friends and family”			.45	*.32*		
“I have principles I live my life by”				.66		
“My past experiences have helped me learn about life”				.59		
“I understand the realities of life”				.49		
“I can always present myself in the way I want to”					.92	
“I have no problems taking care of the place where I live”					.42	
“I can always keep my mind on what I’m doing”						.63
“I can always think of ways to solve my problems”				*.31*		*.39*

*Note*: Italics indicate items with low factor loading (<.40) on the target latent variable.

Three items (“I can see the funny side of life,” “I have things to look forward to,” and “I can always think of ways to solve my problems”) did not load onto any of the six factors. We retained these items on the factor onto which the item loaded most strongly, as, after sensitivity analysis, it was determined that removal of any of the three items was not found to significantly improve the fit of the model (Table 4). In each case, the item fitted conceptually with the factor.

**Table 4.** Sensitivity Analysis of the IDoR Subscale

Model fit indices	Item removed			
	None	“I can see the funny side of life”	“I have things to look forward to”	“I can always think of ways to solve my problems”
TLI	.922	.93	.943	.924
Cronbach’s α	.890	.885	.883	.884
Explained variance	.56	.57	.56	.56

*Note*: IDoR = individual determinants of resilience; TLI = Tucker–Lewis Index.

### Validity and Consistency of the IDoR Subscale

Concerning discriminant validity, the factor correlation matrix showed no correlations greater than .7 (range: .19–.63; Supplementary Table A), with the largest correlation being between the factors “Interpersonal skills” and “Self-care ability,” thus implying that each factor assesses a unique construct.

The Cronbach’s α value of the IDoR subscale was .89 (Supplementary Table C), indicating that the IDoR subscale is internally consistent and reliable (de Vaus, 2002). Mean inter-item correlations within factors were also satisfactory (Tavakol & Dennick, 2011), ranging between .331 and .492 (Supplementary Table B).

All items in the IDoR subscale demonstrated good item discrimination values (range: .366–.632; Nunnally & Bernstein, 1994), while item difficulty values ranged between .48 and .83 (Supplementary Table C). These results indicate that the items in the IDoR subscale are effective in differentiating between those with high IDoR and those with poorer IDoR, thus supporting the reliability of the subscale.

### EFA of the EDoR Subscale

EFA with promax rotation was performed on the data to explore the structure of the EDoR subscale. The data were deemed suitable for EFA based on a KMO value of .84 (Kaiser, 1974) and Bartlett’s test of sphericity which indicated that correlations between items were sufficient (χ ^2^ (78) = 2257.224 [*p* < .001]).

Parallel analysis suggested that five factors should be extracted, while inflexions in the scree plot suggested five or six factors (Supplementary Figure D). Accordingly, the loadings of five- and six-factor solutions were estimated and examined.

The six-factor solution yielded parameter estimates out with the permissible range (factor loadings > 1). In comparison, the five-factor solution had a TLI value of .936 and yielded well-defined and theoretically interpretable factors. Based on the content of high loading items, these factors were labeled “Person–environment fit,” “Friends,” “Material assets,” “Habits,” and “Family” (Table 5). This model accounted for 59% of the common variance, and no items were found to cross-load.

**Table 5.** Five-Factor Solution for the “Environmental Determinants of Resilience” Subscale

Item	Factor 1	Factor 2	Factor 3	Factor 4	Factor 5
	Person–environment fit	Friends	Material assets	Habits	Family
“I can take part in the leisure activities that I want”	.75				
“I have additional roles in my community/society”	.72				
“I can take part in the social activities that I want”	.71				
“I can find and use the learning/training resources I want”	.64				
“I have no problems getting around my home and neighborhood”	.44				
“I can find and use community services I need”	.40				
“I am part of a circle of friends”		.97			
“My circle of friends helps me get through life’s demands”		.66			
“I live in safe and suitable housing”			.64		
“I can afford the things that I need”			.63		
“I am always satisfied with my daily routine”				.86	
“I have no problems organizing my routine so that I can do the things that are important to me”				.68	
“I have family who support me”					.68

### Validity and Consistency of the EDoR Subscale

Concerning discriminant validity, the factor correlation matrix showed no correlations greater than .59 (range: .8–.59) (Supplementary Table D), with the largest correlation being between the factors “Person–environment fit” and “Habits.”

The Cronbach’s α value of the EDoR subscale was .82 (Supplementary Table F), demonstrating good internal consistency (de Vaus, 2002). Omitting the item titled “I have family who support me” would increase the α value, however not substantially (by .001). Removing any other item would cause α to decrease (Supplementary Table F). Mean inter-item correlations within factors ranged between .308 and .683 (Supplementary Table E).

All items in the EDoR subscale demonstrated satisfactory item discrimination values (range: .21–.681), while item difficulty values ranged between .35 and .84 (Supplementary Table F). These results indicate that the items in the EDoR subscale are effective in differentiating between those with high EDoR and those with poorer EDoR.

As the item “I have family who support me” was found to have moderate discrimination effectiveness and given the Cronbach’s α would increase with its removal, the EFA was repeated without this item included. However, removal of this item resulted in parameter estimates out with the permissible range (factor loading > 1). Furthermore, the support of families is of theoretical importance when considering the resilience of older adults. Consequently, this item was retained in the EDoR subscale.

### Correlation Between Subscales

The relationship between the two subscales was assessed using a Pearson’s product–moment correlation coefficient. The two subscales were found to be strongly correlated (*r*(416) = .71 [*p* < .001]) (de Vaus, 2002).

### Convergent Validity

Table 6 displays the unstandardized regression coefficients (*B*) between the IDoR and EDoR scores and the variables of frailty, perceived physical health, and perceived mental health. Both IDoR and EDoR were found to be significantly related with perceived physical and mental health.

**Table 6.** Univariable Regression Analysis Between IDoR, EDoR, and Related Variables

Independent variables	Univariable regression estimates	
	IDoR subscale B (95% CI)	EDoR subscale B (95% CI)
Clinical Frailty scale		
Intercept Managing well Vulnerable Mildly frail Moderately frail Severely frail	46.33 (42.06, 50.61)*** Reference 1.63 (−3.06, 6.32) −1.88 (−6.36, 2.61) −4.75 (−9.16, −0.34)* −7.33 (−12.27, −2.39)**	29.83 (27.03, 32.63) *** Reference −2.7 (−5.77, 0.37) −3.94 (−6.87, −1.01)** −6.15 (−9.03, −3.27)*** −6.47 (−9.70, −3.24)***
Optum SF-12v2 Health Survey—PCS		
Intercept PCS	34.04 (31.48, 36.61)*** 0.29 (0.21, 0.37)***	18.27 (16.63, 19.91)*** 0.21 (0.16, 0.26)***
Optum SF-12v2 Health Survey—MCS		
Intercept MCS	27.09 (23.15, 31.03)*** 0.33 (0.25, 0.41)***	16.55 (12.91, 19.19)*** 0.17 (0.12, 0.23)***

*Notes*: CI = confidence interval; IDoR = individual determinants of resilience; EDoR = environmental determinants of resilience; PCS = Physical Component Summary; MCS = Mental Component Summary.

*p < .05. **p < .01. ***p < .005.

Increasing frailty (reference: managing well) was found to have an increasingly negative effect on IDoR and EDoR. Significant associations were seen in the higher CFS categories, where being mildly frail was associated with decreased EDoR score, and being moderately frail and severely frail associated with both a decreased IDOR and EDoR score, when compared to managing well.

## Discussion

Previous resilience research conducted with older adults has used measures which fail to consider the EDoR and has predominantly focused on community-dwelling older adults. Moreover, this research has paid more attention to protective and vulnerability factors within the older adults than within their community or relationships (Table 1). As a result, the resilience of hospitalized older adults and the environmental determinants of older adults’ resilience have received less attention. This study was undertaken to assess the validity of the MiC questionnaire, a measure of the individual and environmental determinants of older adults’ resilience, with a population of older adults ready for discharge from a MoE ward.

EFA was conducted to examine the construct validity of the MiC questionnaire subscales, item analysis was conducted to assess the quality of the subscales’ items, and regression analysis was conducted to assess the convergent validity of the IDoR and EDoR subscales.

### IDoR Subscale

Six factors were within the IDoR subscale: (1) Self-efficacy, (2) Values, (3) Interpersonal skills, (4) Life orientation, (5) Self-care ability, and (6) Process skills. Cronbach’s α indicated that the IDoR subscale is internally consistent, while item analysis techniques demonstrated that the IDoR subscale items have acceptable discrimination effectiveness.

Comparing these results with existing research offers preliminary support for the construct validity of the MiC questionnaire, as the factors reflect characteristics which have been found to be present in resilient individuals. In addition, the results of the regression analyses support the validity of the IDoR subscale, as they echo the findings of previous resilience research which has found comparable relationships between resilience and similar variables (e.g., physical health: Hildon et al. [2008] and Jeste et al. [2019]; mental health: Lamond et al. [2008] and Liddell and Ferreira [2019]; perceived health: Hardy et al. [2004]).

Three items were found to load poorly onto their respective factors (factor loading < .4); however, removal of any of these items would result in theoretically important information being lost as each item taps into a unique quality of a resilient individual, specifically their sense of humor (Earvolino-Ramirez, 2007), hope, and optimism for the future (Martin et al., 2015; Polson et al., 2018), and adaptability and ability to solve problems when they arise (Earvolino-Ramirez, 2007). As such, the items were retained in the subscale.

### EDoR Subscale

Five factors were found in the EDoR subscale: (1) Person–environment fit, (2) Friends, (3) Material assets, (4) Habits, and (5) Family.

The inclusion of an EDoR subscale in the MiC questionnaire is a particular strength of the questionnaire, as it is recognized that previous resilience measures often overlook the role of environmental factors in determining an individual’s resilience (Windle et al., 2011). Unfortunately, this makes it difficult to compare the items and factors of this subscale with those of another resilience measure. Nevertheless, the factors identified do broadly reflect environmental factors that have been found to relate to older adults’ resilience in existing literature (see Table 1), and significant relationships were found between EDoR score and variables known to be associated with resilience (see Table 6).

As with the IDoR subscale, Cronbach’s α indicated that the EDoR subscale is internally consistent, while item analysis techniques demonstrated that the EDoR subscale items have acceptable discrimination effectiveness. However, the item “I have family who support me” was found to slightly reduce the Cronbach’s α value, and only demonstrated moderate item discrimination. Moreover, this item was the sole item in the “family factor.”

Nevertheless, the item was retained as sensitivity analysis found that its removal would result in other item parameter estimates having factor loadings greater than 1, and would only minimally improve in the Cronbach’s α value. Furthermore, family support is a recognized protective factor of the resilience of older adults (McKibbin et al., 2016; Wells, 2010) and is understood to have a unique role on resilience when compared to social support from friends (Gouveia et al., 2016)—this is supported by the EFA, which found that the items concerning friends formed a distinct factor.

### Limitations of the MiC Questionnaire

Retaining the poorly loading items in each subscale meant that theoretically important information was not lost. However, the resulting factor solutions consisted of multiple factors including only a few items, thus impacting the psychometric properties of the subscales.

One solution to this would be to reduce the number of factors extracted in the EFA. Yet, in this analysis, the number of factors extracted resulted in theoretically interpretable results, whereas a reduced factor solution would have caused factors to contain items with disparate themes, reducing the interpretability of results (Worthington & Whittaker, 2006). For this reason, the original factor structures were retained.

An alternative solution would be to add more items that represent these factors to more robustly capture that dimension. However, the MiC questionnaire consists of 34 items and demonstrates psychometric properties consistent with assessment tools of a similar length (e.g., Gosling et al., 2003). Accordingly, it is recommended that the current version of the MiC questionnaire should be used to measure broadly across the factors, in order to provide a profile of resilience at the point of discharge from a MoE ward and inform resilience interventions, service developments, and service planning.

### Study Implications and Recommendations

The study findings have several implications. First, through the validation of the MiC questionnaire, this study raises awareness of the multidimensional influences on older adults’ resilience. Such awareness may enable clinicians to identify older adults who would struggle to “adapt well” following acute hospital admission, thus supporting complex decision making and customized management (Hardy et al., 2004; Hayman et al., 2017; Hicks & Conner, 2014). Given that the MiC questionnaire was originally developed in the community (QMU and NHS Lothian, 2015), and the majority of the participants recruited in this study were about to be discharged back to private residences (78.84%; Table 2), the results of this study also suggest that its validity may be generalizable to a community-dwelling population. However, confirmatory factor analysis with a sample of older adults recruited in the community would verify this.

Recognizing the determinants of older adults’ resilience may also enable the development of evidence-based resilience interventions. It is suggested that occupational therapists may be in a unique position to provide interventions that improve the resilience of older adults given their view of daily activity, including its interpersonal and environmental components, to enable maximum adaptation in the face of difficulty and change (Pozzi et al., 2020). The correlation between the two subscales further supports this recommendation, as the findings suggest that there are interactions between IDoR and EDoR. Therefore, targeting the environmental resources of older adults may also improve their IDoR, and vice versa.

Second, through the recruitment of older adults approaching hospital discharge, this study supports the use of the MiC questionnaire within acute hospital settings, where consideration of older adult’s resilience at discharge may support improvement in patient outcomes (Rebagliati et al., 2016). Nevertheless, a 34-item measure may be difficult to routinely implement at the point of discharge from a busy hospital ward. Consequently, it would be beneficial for future research to assess the validity of individual MiC questionnaire items in predicting negative outcomes following hospital discharge. This would enable researchers to develop a shorter screening tool that could identify older adults who would benefit from resilience interventions, and would make it more applicable for a busy hospital setting where clinicians may be faced with a stark choice of using a brief measure or using no measure at all (Gosling et al., 2003).

In particular, it would be pertinent for this research to assess the ability of the items to predict hospital readmission within 6 months of initial discharge, given that hospital admission is considered a health risk for older adults and up to 50% of older adults discharged from acute hospital care are readmitted within 6 months (de Man et al., 2019).

## Conclusion

This study sought to assess the validity of the MiC questionnaire for use with older adults approaching discharge from a MoE ward. EFA demonstrated that the IDoR and EDoR subscales of the MiC questionnaire reflect current conceptualizations of older adults’ resilience, while regression analyses supported their convergent validity. Cronbach’s α verified the internal consistency of the subscales, while item analysis techniques supported their discrimination effectiveness.

However, multiple factors were found to consist of only one or two items. Nevertheless, it is suggested that the current iteration of the MiC questionnaire should be used to profile the resilience needs of older adults at the point of hospital discharge in order to develop resilience interventions that support older adults’ transition from hospital to home.

Future research should focus on identifying items of the MiC questionnaire which predict hospital readmission in order to develop a screening tool which may be more easily applied to clinical care. Confirmatory factor analysis of the MiC questionnaire could also be conducted with a sample of community-dwelling older adults to support its use in community care settings.

## Supplementary Material

igab030_suppl_Supplementary_Material
