# Supplementary material for: Psychometric Evaluation of the Making it CLEAR Questionnaire: A Resilience Measure for Older Adults
Source: Innov Aging. 2021 Aug 13;5(3):igab030. doi: 10.1093/geroni/igab030 (PMC8528023; doi:10.1093/geroni/igab030)
Supplement: igab030_suppl_Supplementary_Material [file igab030_suppl_supplementary_material.docx]

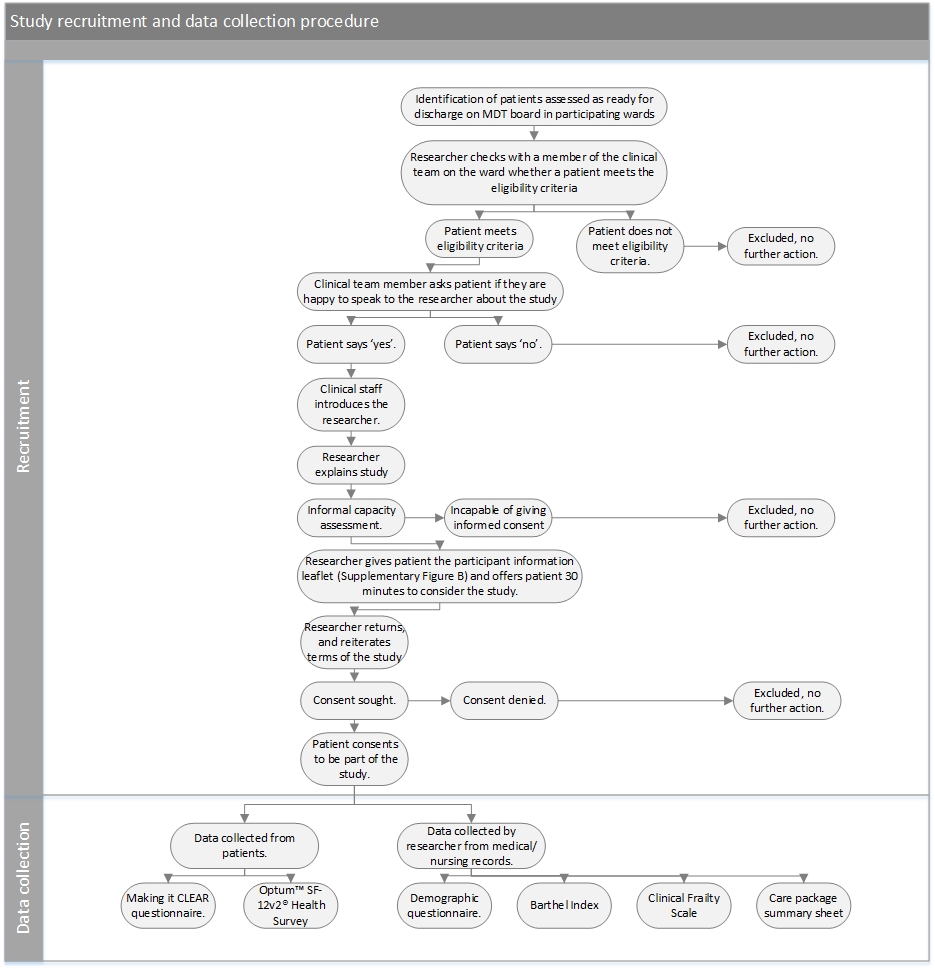


Figure A. Recruitment and data collection procedure for the MiC-MoE study.


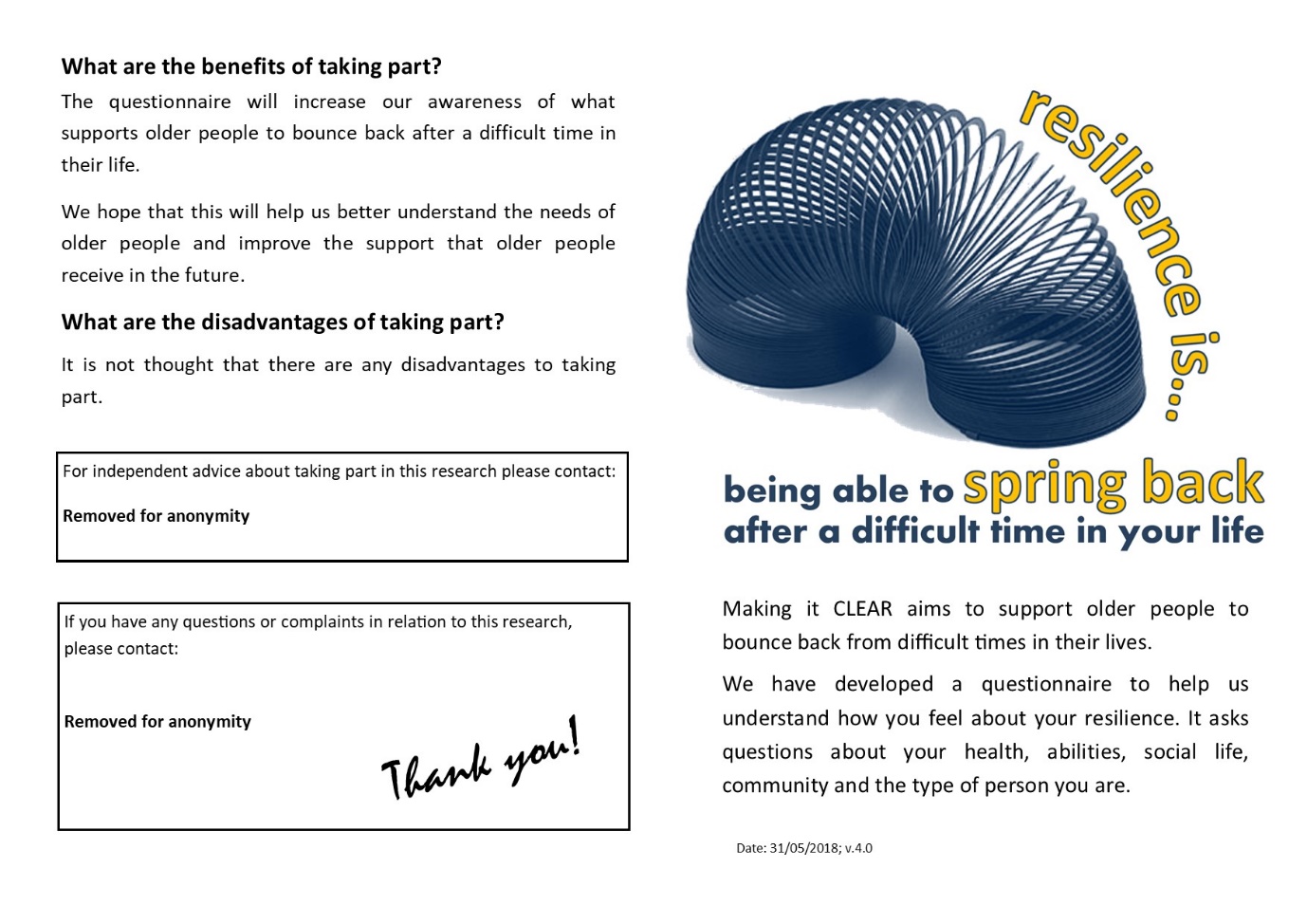


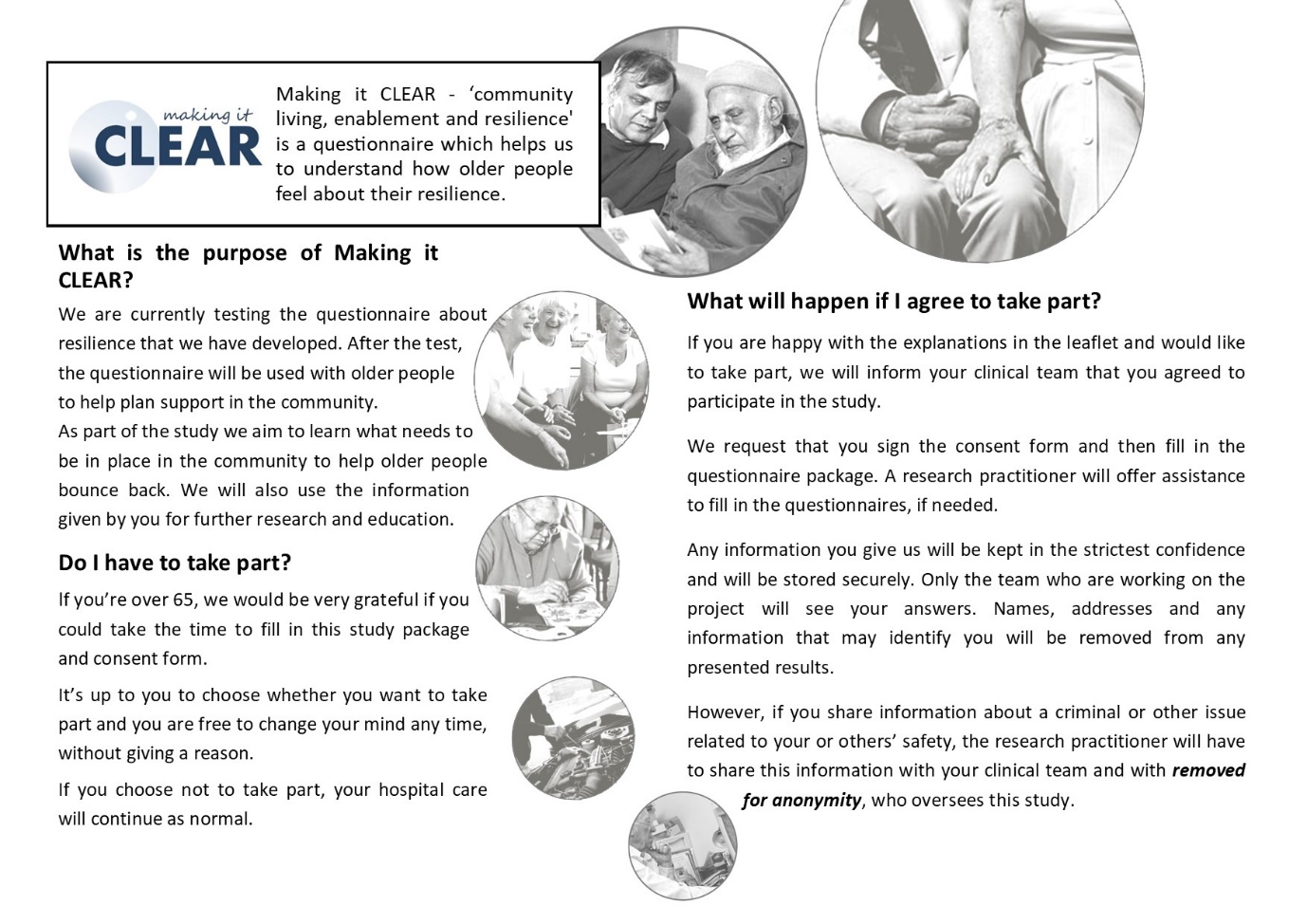


Figure B. Participant information leaflet.


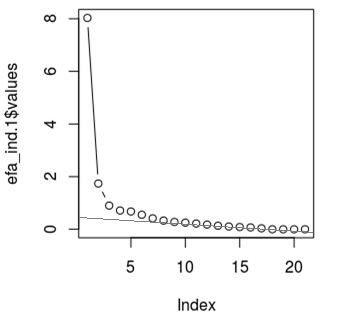


Figure C. Individual Determinants of Resilience Scree Plot.

Table A. Factor correlations of IDoR subscale.

| **FACTORS** | **1** | **2** | **3** | **4** | **5** | **6** |
| --- | --- | --- | --- | --- | --- | --- |
| Self-efficacy | 1 |  |  |  |  |  |
| Values | 0.51 | 1 |  |  |  |  |
| Interpersonal skills | 0.58 | 0.58 | 1 |  |  |  |
| Life orientation | 0.38 | 0.53 | 0.61 | 1 |  |  |
| Self-care ability | 0.61 | 0.41 | 0.63 | 0.49 | 1 |  |
| Process skills | 0.36 | 0.29 | 0.27 | 0.33 | 0.19 | 1 |

Table B. Mean inter-item correlations within IDoR factors.

| FACTOR | MEAN INTER-ITEM CORRELATION |
| --- | --- |
| Self-efficacy | 0.492 |
| Values | 0.348 |
| Inter-personal skills | 0.367 |
| Life orientation | 0.331 |
| Self-care ability | 0.387 |
| Process skills | 0.448 |

Table C: Item analysis of the IDoR subscale.

| ITEM | DESCRIPTIVE STATISTICS AND ITEM ANALYSIS OF THE IDoR SUBSCALE | | | | |
| --- | --- | --- | --- | --- | --- |
|  | **MEAN** | **SD** | **ITEM DIFFICULTY** | **ITEM DISCRIMINATION** | **α IF DELETED** |
| ‘I can always present myself in the way I want to’ | 2.15 | 0.52 | 0.72 | 0.470 | 0.886 |
| ‘I have no problems taking care of the place where I live’ | 1.91 | 0.61 | 0.64 | 0.412 | 0.888 |
| ‘I am a patient person’ | 1.9 | 0.82 | 0.63 | 0.366 | 0.890 |
| ‘I find it easy to accept whatever life throws at me’ | 1.96 | 0.69 | 0.65 | 0.537 | 0.884 |
| ‘I can forgive myself and others’ | 2.19 | 0.55 | 0.73 | 0.438 | 0.887 |
| ‘I have things to look forward to’ | 2.01 | 0.73 | 0.67 | 0.566 | 0.883 |
| ‘I can see the funny side of life’ | 2.4 | 0.68 | 0.80 | 0.524 | 0.885 |
| ‘I have principles I live my life by’ | 2.31 | 0.62 | 0.77 | 0.435 | 0.887 |
| ‘My past experiences have helped me learn about life’ | 2.49 | 0.56 | 0.83 | 0.421 | 0.887 |
| ‘I am happy to help my friends and family’ | 2.49 | 0.55 | 0.83 | 0.452 | 0.887 |
| ‘I am generally happy’ | 2.23 | 0.62 | 0.74 | 0.620 | 0.882 |
| ‘I understand the realities of life’ | 2.33 | 0.59 | 0.78 | 0.441 | 0.887 |
| ‘I see myself as a healthy person’ | 1.63 | 0.8 | 0.54 | 0.446 | 0.887 |
| ‘I am able to do things on my own’ | 1.91 | 0.79 | 0.64 | 0.571 | 0.883 |
| ‘I feel in control of my life’ | 1.91 | 0.73 | 0.64 | 0.632 | 0.881 |
| ‘I am physically able to do the things I need and want to’ | 1.58 | 0.83 | 0.53 | 0.573 | 0.883 |
| ‘I always have enough energy to do the things I need and want to’ | 1.43 | 0.79 | 0.48 | 0.573 | 0.883 |
| ‘I can always make myself understood to others’ | 2.19 | 0.64 | 0.73 | 0.491 | 0.886 |
| ‘I have no problems getting along with others and making new friends’ | 2.29 | 0.58 | 0.76 | 0.498 | 0.885 |
| ‘I can always keep my mind on what I am doing’ | 1.94 | 0.69 | 0.65 | 0.488 | 0.886 |
| ‘I can always think of ways to solve my problems’ | 1.98 | 0.64 | 0.66 | 0.537 | 0.884 |
|  | | | | | |
|  | | | | **Cronbach’s α** | 0.890 |


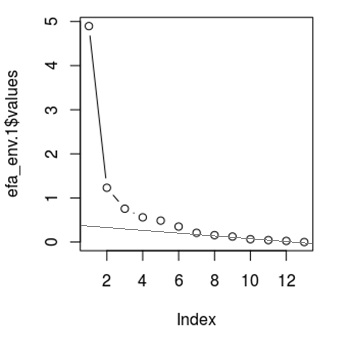


Figure D. Environmental Determinants of Resilience Scree Plot.

Table D: Factor correlations in EDoR subscale.

| **FACTORS** | **1** | **2** | **3** | **4** | **5** |
| --- | --- | --- | --- | --- | --- |
| Person-environment fit | 1 |  |  |  |  |
| Friends | 0.57 | 1 |  |  |  |
| Material Assets | 0.50 | 0.27 | 1 |  |  |
| Habits | 0.59 | 0.31 | 0.59 | 1 |  |
| Family | 0.08 | 0.10 | 0.22 | 0.17 | 1 |

Table E: Mean inter-item correlations within EDoR factors.

| FACTOR | MEAN INTER-ITEM CORRELATION |
| --- | --- |
| Person-environment fit | 0.335 |
| Friends | 0.683 |
| Material assets | 0.308 |
| Habits | 0.591 |
| Family | - |

*Note.* The inter-item correlation for the factor ‘family’ could not be calculated due to it consisting of only one item.

Table F. Item analysis of the EDoR subscale.

| ITEM | DESCRIPTIVE STATISTICS AND ITEM ANALYSIS OF THE EDoR SUBSCALE | | | | |
| --- | --- | --- | --- | --- | --- |
|  | **MEAN** | **SD** | **ITEM DIFFICULTY** | **ITEM DISCRIMINATION** | **α IF DELETED** |
| ‘I can take part in the leisure activities that I want’ | 1.56 | 0.78 | 0.52 | 0.544 | 0.804 |
| ‘I can take part in the social activities that I want’ | 2.01 | 0.71 | 0.67 | 0.681 | 0.793 |
| ‘I can find and use the community services I need’ | 2.04 | 0.57 | 0.68 | 0.393 | 0.816 |
| ‘I can find and use the learning/training resources that I want’ | 1.74 | 0.69 | 0.58 | 0.475 | 0.810 |
| ‘I have additional roles in my community/society’ | 1.06 | 0.67 | 0.35 | 0.402 | 0.815 |
| ‘I am part of a circle of friends’ | 1.76 | 0.84 | 0.59 | 0.559 | 0.802 |
| ‘I have no problems getting around my home and neighbourhood’ | 1.79 | 0.79 | 0.60 | 0.484 | 0.809 |
| ‘I live in safe and suitable housing’ | 2.53 | 0.54 | 0.84 | 0.404 | 0.815 |
| ‘My circle of friends helps me get through life’s demands’ | 1.75 | 0.84 | 0.58 | 0.529 | 0.805 |
| ‘I have family who support me’ | 2.53 | 0.77 | 0.84 | 0.210 | 0.832 |
| ‘I can afford the things that I need’ | 2.33 | 0.54 | 0.78 | 0.297 | 0.821 |
| ‘I am always satisfied with my daily routine’ | 1.83 | 0.67 | 0.61 | 0.483 | 0.809 |
| ‘I have no problems organising my routine so that I can do the things that are important to me’ | 2.03 | 0.62 | 0.68 | 0.582 | 0.803 |
|  | | | | | |
|  | | | | **Cronbach’s α** | **0.823** |
